# Ubiquitin-Specific Protease 2 Modulates the Lipopolysaccharide-Elicited Expression of Proinflammatory Cytokines in Macrophage-like HL-60 Cells

**DOI:** 10.1155/2017/6909415

**Published:** 2017-09-12

**Authors:** Hiroshi Kitamura, Takeshi Ishino, Yoshinori Shimamoto, Jun Okabe, Tomomi Miyamoto, Eiki Takahashi, Ichiro Miyoshi

**Affiliations:** ^1^Laboratory of Veterinary Physiology, Department of Veterinary Medicine, School of Veterinary Medicine, Rakuno Gakuen University, 582 Bunkyodai-Midorimachi, Ebetsu, Hokkaido 069-8501, Japan; ^2^Department of Comparative and Experimental Medicine, Graduate School of Medical Sciences, Nagoya City University, Nagoya, Aichi, Japan; ^3^Laboratory of Animal Therapeutics, Department of Veterinary Science, Rakuno Gakuen University, 582 Bunkyodai-Midorimachi, Ebetsu, Hokkaido 069-8501, Japan; ^4^Department of Diabetes, Central Clinical School, Faculty of Medicine, Nursing and Health Sciences, Monash University, 99 Commercial Road, Melbourne, VIC 3004, Australia; ^5^Research Resources Center, RIKEN Brain Science Institute, 2-1 Hirosawa, Wako, Saitama, Japan

## Abstract

We investigated the regulatory roles of USP2 in mRNA accumulation of proinflammatory cytokines in macrophage-like cells after stimulation with a toll-like receptor (TLR) 4 ligand, lipopolysaccharide (LPS). Human macrophage-like HL-60 cells, mouse macrophage-like J774.1 cells, and mouse peritoneal macrophages demonstrated negative feedback to USP2 mRNA levels after LPS stimulation, suggesting that USP2 plays a significant role in LPS-stimulated macrophages. *USP2* knockdown (KD) by short hairpin RNA in HL-60 cells promoted the accumulation of transcripts for 25 of 104 cytokines after LPS stimulation. In contrast, limited induction of cytokines was observed in cells forcibly expressing the longer splice variant of USP2 (USP2A), or in peritoneal macrophages isolated from *Usp2a* transgenic mice. An ubiquitin isopeptidase-deficient USP2A mutant failed to suppress LPS-induced cytokine expression, suggesting that protein ubiquitination contributes to USP2-mediated cytokine repression. Although USP2 deficiency did not accelerate TNF receptor-associated factor (TRAF) 6-nuclear factor-*κ*B (NF-*κ*B) signaling, it increased the DNA binding ratio of the octamer binding transcription factor (Oct)-1 to Oct-2 in *TNF*, *CXCL8*, *CCL4*, and *IL6* promoters. USP2 decreased nuclear Oct-2 protein levels in addition to decreasing the polyubiquitination of Oct-1. In summary, USP2 modulates proinflammatory cytokine induction, possibly through modification of Oct proteins, in macrophages following TLR4 activation.

## 1. Introduction

Inflammation is the basis for a wide variety of diseases. In addition to canonical inflammatory diseases, such as inflammatory bowel disease and septic shock, it also constitutes a pathological basis for atherosclerosis, type 2 diabetes, and carcinogenesis [1–3]. In inflammatory responses, macrophages are the predominant effector cells in terms of number and cellular function. They abundantly express toll-like receptors (TLRs), which recognize and bind to specific pathogen-associated molecular patterns (PAMPs). In the vertebrate genome, there are 10–15 TLR genes encoding surface and intracellular TLR proteins [4]. Of the surface TLRs, TLR4 responds to bacterial lipopolysaccharide (LPS) and participates in antibacterial immunity [4, 5] by associating with adaptor proteins, such as myeloid differentiation primary response 88 (MyD88) and tumor necrosis factor receptor-associated factor (TRAF) 6, leading to activation of nuclear factor-*κ*B (NF-*κ*B) [4, 6]. This causes macrophages to undergo metabolic reprogramming and subsequently secrete proinflammatory cytokines, such as interleukin (IL)-6, IL-8 (C-X-C motif ligand (CXCL) 8), and tumor necrosis factor (TNF)-*α* [4, 6, 7]. These cytokines contribute to the activation of both the innate and the acquired immune system, chemotaxis of leukocytes, induction of hepatic acute phase proteins, and modulation of hypothalamus function [8–10].

In addition to NF-*κ*B, several transcriptional factors and chromatin modifiers orchestrate transcriptional events during bacterial-induced inflammation [11, 12]. Octamer binding transcription factor (Oct)-1 and Oct-2 belong to the Pit-Oct-Unc (POU) domain transcription factor family. Although they preferably bind to the ATTTGCAT “octamer” motif in *in vitro* models [13], a genome-wide chromatin precipitation (ChIP) sequence analysis showed that Oct-1 and Oct-2 accumulation at the promoter/enhancer is not restricted to the octamer motif [14]. Oct-1 and Oct-2 have been shown to act as both transcriptional activators and repressors, depending on the interacting proteins. For example, Oct-1 activates snRNA transcription by cooperating with SNAPc [15], whereas the Oct-1 and Oct-2 heterocomplex represses transcription of inducible nitric oxide synthase (iNOS) [16].

Ubiquitination and deubiquitination are reversible chemical processes that regulate the molecular properties of target proteins [17]. Deubiquitination is catalyzed by groups of proteases, which remove ubiquitin or ubiquitin-like proteins from the target proteins. The ubiquitin-specific protease (USP) family is the largest group of ubiquitin proteases in the mammalian genome [18]. Increasing evidence suggests that several USPs participate in the modulation of immune and inflammatory signaling [19–21].

USP2 participates in various cellular events, including circadian rhythm modulation [22], carcinogenesis [23], prevention of insulin resistance [24], and spermatogenesis [25]. USP2 encodes two splice variants in humans and mice: the longer splice variant USP2A (approximately 69 kDa) and the shorter USP2B (approximately 45 kDa) [26]. Although both variants share a common C-terminal ubiquitin isopeptidase region, the structure of the N-terminal extension differs in terms of sequence, suggesting distinct roles in cellular processes [27–29]. Previously, He et al. [30] reported that USP2A negatively regulates NF-*κ*B-dependent induction of *IL6* and *TNF* in HCT116 colorectal carcinoma cells. Conversely, Sun et al. [31] reported that USP2 modifies degradation of TNF-*α* protein in macrophages. Although these reports suggest modulatory roles of USP2 in inflammatory responses, its role in cytokine induction in macrophages has not been comprehensively evaluated. In this study, we performed systemic monitoring of cytokine expression in USP2-modified macrophage-like cells, whereby USP2 represses a large number of cytokines after induction by LPS. We also suggest that the deubiquitination of Oct-1 transcription factors by USP2 is involved in the transcriptional regulation of cytokine genes.

## 2. Materials and Methods

### 2.1. Reagents

LPS (*E. coli*, serotype 055:B5; TLR4 ligand) was purchased from Sigma-Aldrich (St. Louis, MO). Pam3CSK4 (TLR1/2 ligand), poly(I:C) (TLR3 ligand), and ODN1826 (TLR9 ligand) were purchased from InvivoGen (San Diego, CA).

### 2.2. Cells

Human myeloid leukemia HL-60, mouse macrophage-like J774.1, and HEK293 FT cells were obtained from the RIKEN BioResource Center (Tsukuba, Japan), the American Type Culture Collection (Manassas, VA), and Thermo Fisher Scientific (Waltham, MA), respectively. The generation of HL-60-derived *USP2*KD cells and their control cells have been described previously [29]. The *USP2*KD-derived cells, in which USP2A (USP2AR), USP2B (USP2BR), and isopeptidase-mutant USP2A (C276AR) were reintroduced, have also been previously discussed [29]. HL-60 cells and their derivatives were differentiated into macrophage-like cells by treatment with 30 nM of phorbol 12-myristate 13-acetate (PMA, Sigma-Aldrich) for 1 d. Peritoneal macrophages were collected from mice treated with thioglycollate medium (2 mL/head; Sigma-Aldrich). Cells were collected from mouse peritoneal cavities using a 10 mL syringe with an 18-gauge needle; after which, macrophages were separated using an adherence-based method [32].

### 2.3. Mice

C57BL/6 mice were purchased from Japan SLC (Shizuoka, Japan). C57BL/6-background *Usp2a* transgenic (Tg) mice have been documented previously [29]. The *Usp2a* transgene was transcribed under the control of the *c-fms* gene intronic element (*FIRE*), which yields abundant expression in macrophages [33]. Male mice 8–10 weeks of age were used in strict accordance with recommendations from the Guide for the Care and Use of Laboratory Animals of the National Institute of Health. The protocol was approved by the Animal Ethics Committees of Nagoya City University (permit number: H22M-54) and Rakuno Gakuen University (permit number: VH16A15).

### 2.4. Quantitative Reverse Transcription-Polymerase Chain Reaction (qRT-PCR)

Total RNA extracted with TRIzol reagent (Thermo Fisher Scientific) was subjected to qRT-PCR as described previously [34]. A quantitative PCR (qPCR) reaction was performed with the KAPA SYBR FAST qPCR Master Mix (KAPA Biosystems, Wilmington, MA) and Platinum Quantitative PCR SuperMix (Thermo Fisher Scientific) using an ECO qPCR system (Illumina, San Diego, CA). TaqMan probe and primer mixtures for human *USP2A* and *USP2B* were purchased from Thermo Fisher Scientific. The qPCR primer and probe sequences are listed in Table 1.

### 2.5. Enzyme-Linked Immunosorbent Assay (ELISA)

Supernatants of HL-60 cells and their derivatives were stored at −80°C until use. The IL-6 concentration was determined using an ELISA kit purchased from BioLegend (San Diego, CA).

### 2.6. Western Blot Analysis

Total cell lysates were prepared with RIPA buffer (Nacalai Tesque, Kyoto, Japan). Nuclear and cytoplasmic protein was extracted using a Nuclear Extraction Kit (Active Motif, Carlsbad, CA). After electrophoresis in a 10% SuperSep gel (Wako Chemicals, Osaka, Japan), protein was transferred onto an Immobilon-P PVDF membrane (EMD Millipore, Billerica, MA) and blocked with Blocking-One solution (Nacalai Tesque). For Western blot analysis, a 1000-fold dilution of antibodies against USP2 (AP2131a and AP2131c; Abgent, San Diego, CA), TRAF6 (ab33915; Abcam, Cambridge, UK), RelA (sc-372; Santa Cruz Biotechnology (SCB), Dallas, TX), p50 (sc-1190; SCB), I*κ*B*α* (#9242; Cell Signaling Technology (CST), Danvers, MA), Oct-1 (A301-717A; Bethyl Laboratories, Montgomery, TX), Oct-2 (ab179808; Abcam), Oct-6 (sc-390056; SCB), GAPDH (sc-32233; SCB), and lamin-A/C (sc-6215; SCB) was used as primary antibodies. A 2000-fold diluted horseradish peroxidase-conjugated anti-rabbit (#7074; CST) or anti-goat (sc-2056; SCB) immunoglobulins were used as the secondary antibodies. Primary and secondary antibodies were reacted with the Western blot membranes in Hikari enhancer solutions (Nacalai Tesque). Chemiluminescent signals were visualized using Chemilumi One Super reagent (Nacalai Tesque) and scanned using an EzCapture system (Atto, Tokyo, Japan). Intensities of immune signals were digitalized using a CS Analyzer 3.0 program (Atto).

### 2.7. Chromatin Accessibility by Real-Time PCR (CHART-PCR)

CHART-PCR was conducted following the previous reports [35]. Briefly, cells were lysed in a buffer containing 10 mM Tris (pH 7.4), 10 mM NaCl, 3 mM MgCl_2_, 0.5% Nonidet P-40, 150 *μ*M spermine, and 500 *μ*M spermidine. After centrifuging the cell extract in a wash buffer (10 mM Tris (pH 7.4), 15 mM NaCl, 60 mM KCl, 150 *μ*M spermine, and 500 *μ*M spermidine), the nuclear pellet was resuspended in a digestion buffer (10 mM Tris (pH 7.4), 15 mM NaCl, 60 mM KCl, 150 *μ*M spermine, 500 *μ*M spermidine, and 1 mM CaCl_2_) with or without micrococcal nuclease (10 U/mL; Takara Bio, Otsu, Japan) at 37°C for 30 min. Subsequently, the reaction was stopped by adding 30 mM EDTA, 3 mM EGTA, 0.75% SDS, and 350 *μ*g/mL proteinase K (New England Bio Labs, Ipswich, MA), followed by incubation at 37°C for 2 h. After phenol-chloroform extraction, DNA was subjected to qPCR analysis. Chromatin accessibility was calculated using the EpiQ chromatin kit data analysis tool (BioRad Laboratories, Hercules, CA) and data from glyceraldehyde-3-phosphate dehydrogenase (*GAPDH*) and hemoglobin promoters as positive and negative controls. The PCR primer sequences for cytokines and reference genes are shown in Table 2.

### 2.8. Immunoprecipitation-Western Blot Analysis

Immunoprecipitation of TRAF6 was performed using an anti-TRAF6 (ab33915; Abcam) antibody with a Pierce Crosslink IP Kit (Thermo Fisher Scientific) according to the manufacturer's instructions. For detection of Western blot bands, TRAF6 (ab33915; Abcam), polyubiquitin chains formed by K63-linked (ab179434; Abcam) and K48-linked (ab140601; Abcam) primary antibodies were used (1000-fold dilution).

### 2.9. Transcription Factor Beads Array

Multiplex analysis of the DNA binding activity of transcription factors was performed using a Bio-Plex 2000 system (BioRad Laboratories) with a Procarta Transcription Factor Plex Kit (Thermo Fisher Scientific). Data were normalized using a standard quantile normalization method and subsequently visualized using the regHeatmap program in the Bioconductor Heatplus analysis package (https://github.com/alexploner/Heatplus).

### 2.10. Microarray Data

Microarray data of mouse macrophages and macrophage-like cells were obtained from RefDic [36]. Heatmaps of the expression data were illustrated using the expression profile function of RefDic.

### 2.11. Detection of Oct-1 and Oct-2 Binding Sites

Binding sites of Oct-1 and Oct-2 in the 5′ proximal regions of human cytokine genes were screened using the JASPAR (http://jaspar.genereg.net/) and DECODE (http://www.sabiosciences.com/chipqpcrsearch.php) databases. Oct-2-ChIP sequence data sets were also deposited in the ENCODE database (http://www.epigenomebrowser.org/ENCODE/; Accession numbers ENCSR000BGP and ENCSR000BII). The ATTTGCAT octamer motif was searched for within ±2 kb proximal regions of transcriptional initiation sites of the cytokine genes using the UCSC genome browser (http://genome.ucsc.edu/).

### 2.12. ChIP-PCR

Chromatin samples for ChIP were prepared using a Simple ChIP Enzymatic Chromatin IP Kit (CST) and were precleaned with Protein A-conjugated Dynabeads (Thermo Fisher Scientific) at 4°C for 2 h. Antibodies against Oct-1 (ab15112, Abcam) and Oct-2 (ab179808, Abcam) were bound to Protein A-conjugated Dynabeads, then subjected to immunoprecipitation with the precleaned samples at 4°C overnight. Washing beads and eluting immunoprecipitated DNA fragments were prepared according to the CST manual. qPCR was performed as described above. The qPCR primer sequences were determined based on the Oct-1 or Oct-2 binding sites deposited in the JASPAR and DECODE databases. The qPCR primer sequences for ChIP-PCR are listed in Table 3. Data were normalized using the Ct values of input samples.

### 2.13. Pull-Down of Oct-1 and Oct-2

The coding region of human USP2A (accession number NM_004205) without the stop codon was cloned into pcDNA3.2/V5-DEST (Thermo Fisher Scientific) using BP clonase II (Thermo Fisher Scientific). The resultant plasmid encoded C-terminal V5-tagged USP2A. Halo-tagged Oct-1 and Oct-2 expression plasmids were purchased from Promega (Madison, WI). The HA-tagged ubiquitin plasmid was purchased from Addgene (Cambridge, MA). The USP2A, or control pcDNA3.2/V5-DEST plasmid, plasmids encoding Halo-tagged Oct-1 or Oct-2 and HA-tagged ubiquitin were transfected into HEK293FT cells using Lipofectamine 2000 reagent (Thermo Fisher Scientific). The pull-down and elution of Oct proteins were performed using Magne HaloTag beads (Promega) and HaloTEV protease (Promega) as per the manufacturer's instructions. For detection of Western blot bands, corresponding to K48- and K63-linked ubiquitin chains, Oct proteins, HA-tagged ubiquitin, and V5-tagged USP2, 1000-fold-diluted antibodies against polyubiquitin chains formed by K48 residues (ab140601; Abcam) and K63 residues (ab179434; Abcam), Oct-1 (A301-717A; Bethyl Laboratories), Oct-2 (ab179808; Abcam), HA-tag (#3724; CST), and V5-tag (A190-120F; Bethyl Laboratories) were used as the primary antibodies.

### 2.14. Statistical Analysis

Statistical analysis was performed using the Student *t*-test or one-way analysis of variance (ANOVA) followed by the Tukey's post hoc test using the Kaleida Graph software package (Hulinks, Tokyo, Japan).

## 3. Results

### 3.1. LPS and Pam3CSK4 Repress the Expression of USP2 Splice Variants in Macrophages

**Figure 1:**
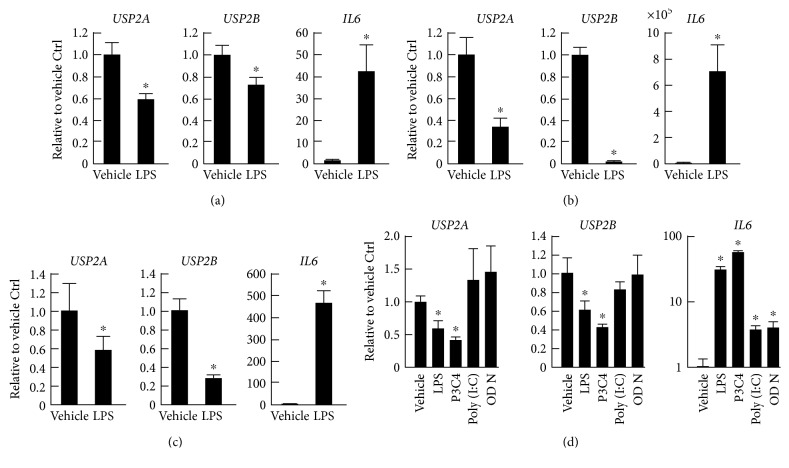
Expression levels of *USP2* and *IL6* in macrophages following TLR ligand stimulation. (a) HL-60 cells were pretreated with PMA (30 nM) for 1 d. (b) J774.1 cells and (c) mouse peritoneal macrophages were treated with LPS (2.5 *μ*g/mL) or vehicle alone for 4 h. (d) PMA-pretreated HL-60 cells were stimulated with LPS (2.5 *μ*g/mL), Pam3CSK4 (P3C4; 5 *μ*g/mL), poly(I:C) (5 *μ*g/mL), ODN1826 (ODN; 2.5 *μ*M), or vehicle alone for 4 h. mRNA levels of *USP2A*, *USP2B*, and *IL6* are normalized according to the level of *HPRT-1* mRNA. Data presented as means ± SD of (a) five, (b) three, or (c and d) four samples. ^∗^*P* < 0.05 versus vehicle-treated control.

We first examined whether proinflammatory stimulation affects the expression of USP2 splice variants in several macrophage-like cell lines and isolated macrophages. Human myeloid HL-60 cells were differentiated into macrophage-like cells in the presence of PMA (30 nM) for 1 d, then stimulated with *E. coli* LPS (2.5 *μ*g/mL) for 4 h. In contrast to *IL6* expression, the mRNA levels of both *USP2A* and *USP2B* significantly decreased as a result of the LPS treatment (Figure 1(a)). LPS also decreased *Usp2a* and *Usp2b* transcripts in the mouse macrophage-like J774.1 cells and peritoneal macrophages (Figures 1(b) and 1(c)), while *IL6* was upregulated in these cells. Similar decreases in *USP2* transcripts were also observed in HL-60 cells after stimulation with the TLR1/2 ligand, Pam3CSK4 (Figure 1(d)). Conversely, the TLR3 ligand, poly(I:C), and TLR9 ligand, ODN1826, did not induce expressional changes in the *USP2* splice variants, although they did increase *IL6* expression. The TLR4 and TLR1/2 ligands, therefore, decreased the expression of *USP2* splice variants in macrophages. This negative feedback on *USP2* mRNA after TLR stimulation suggests that USP2 has certain roles in macrophages during LPS-induced inflammatory states.

### 3.2. *USP2* Knockdown (KD) Promotes LPS-Elicited Cytokine Production in Macrophage-Like HL-60 Cells

**Figure 2:**
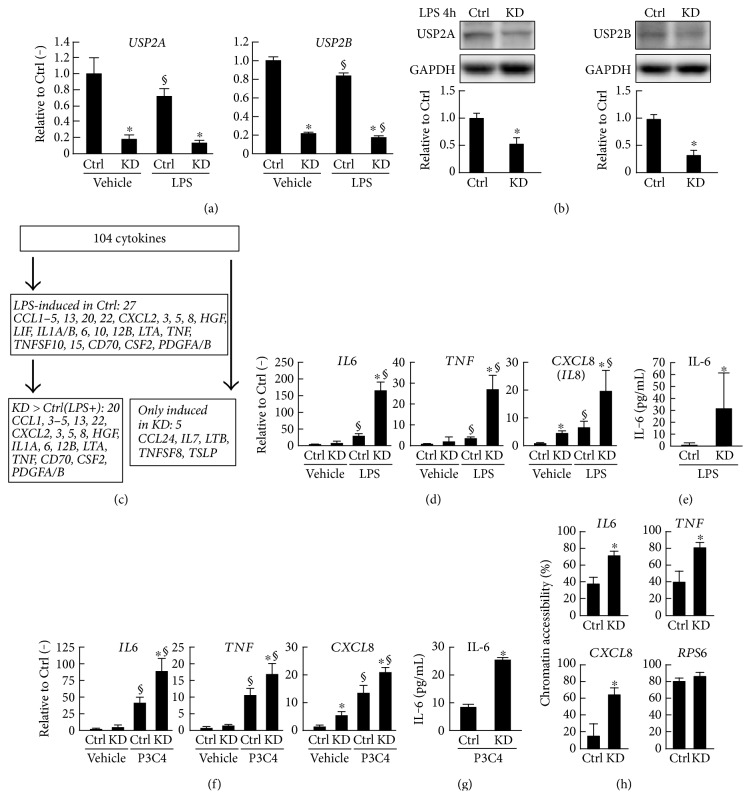
*USP2* knockdown (KD) causes an increase in the expression of proinflammatory cytokines. *USP2*KD (KD) and mock-transfected control (Ctrl) cells were treated with PMA (30 nM; 1 d) and subsequently stimulated with LPS (2.5 *μ*g/mL; a–e, h), Pam3CSK4 (5 *μ*g/mL; f, g), or vehicle alone for (h) 1 h, (a–d, f) 4 h, or (e, g) 12 h. mRNA levels of (a) *USP2* splice variants and (d, f) cytokines were determined by qRT-PCR and normalized according to the level of *HPRT-1* mRNA. (b) Western blot analysis of USP2 protein. Signal intensities of the bands were measured and normalized to the level of GAPDH. Representative images are shown. (c) Schematic summary of the comprehensive cytokine expression analysis. mRNA levels of 104 cytokines were determined by qRT-PCR. Triplicate samples were used in the analysis. (e, g) The IL-6 concentration in the supernatant of *USP2*KD cells was determined by ELISA. (h) Chromatin accessibility of 5′ flanking region of *IL6*, *TNF*, and *CXCL8* in *USP2*KD cells. The *RPS6* gene was used as a positive reference. Data are presented as means ± SD of (a, d, f) six, (b) three, (e, g) eight, or (h) five samples. ^∗^*P* < 0.05 versus control cells; ^§^*P* < 0.05 versus vehicle-treated cells.

After demonstrating that the expression of USP2 is regulated by TLR stimuli in a negative feedback manner, we hypothesized that *USP2* may participate in the TLR ligand-elicited production of proinflammatory cytokines in macrophages. To examine this, we compared the abundance of cytokine transcripts in HL-60 cells transfected with *USP2*-targeting (*USP2*KD) or nontargeting shRNAs. Our results confirmed those of our previous study [29], which suggested that *USP2*KD cells exhibited approximately 80% repression of *USP2* mRNA in parallel with significant decreases in USP2 proteins (USP2A, 53% of control cells; USP2B, 32% of control cells; Figures 2(a) and 2(b)). Next, we measured the abundance of transcripts for 104 cytokines in *USP2*KD and control cells 4 h after LPS or vehicle treatment by comprehensive qRT-PCR (Figure 2(c)). The transcripts for 27 cytokines significantly increased (>1.5-fold, *P* < 0.05) in control cells after LPS stimulation. Of these, 20 cytokines exhibited greater increases in *USP2*KD cells than in control cells. For example, *IL6*, *TNF*, and *CXCL8* (*IL8*) transcripts increased approximately 170-, 28-, and 20-folds, respectively, in LPS-stimulated *USP2*KD cells relative to unstimulated control cells, while the increases in expression were substantially lower in mock-infected cells (approximately 30-, 3-, and 6-folds, resp., Figure 2(d)). Accordingly, the concentration of IL-6 in the supernatant of the *USP2*KD cells was greater than in that of control cells following LPS stimulation (Figure 2(e)). Similarly, *USP2*KD cells exhibited augmented expressions of *IL6*, *TNF*, and *CXCL8* after Pam3CSK4 stimulation, followed by increased IL-6 concentrations in their supernatants (Figures 2(f) and 2(g)). These results show USP2 functions as a global anti-inflammatory modulator in macrophages.

The abundance of cellular mRNA is regulated by both activation of transcription and by repression of mRNA degradation. We next investigated whether USP2 activates transcription of the cytokine genes by modifying chromatin accessibility after TLR stimulation. As shown in Figure 2(h), the chromatin accessibility of the 5′-proximal region of the *IL6* gene was approximately 2-fold greater in *USP2*KD cells than in control cells 1 h after LPS stimulation. Similarly, the accessibility of the proximal regions of *TNF* and *CXCL8* genes was also substantially increased in *USP2*KD cells. Conversely, the accessibility of the ribosomal protein *RPS6* gene was not influenced by *USP2* deficiency. Together, these results indicate that USP2 selectively modulates the transcription of proinflammatory cytokines in response to LPS through conformational changes in the proximal cytokine promoters.

### 3.3. USP2A Represses Proinflammatory Cytokine Expression in HL-60 Cells after LPS Stimulation

**Figure 3:**
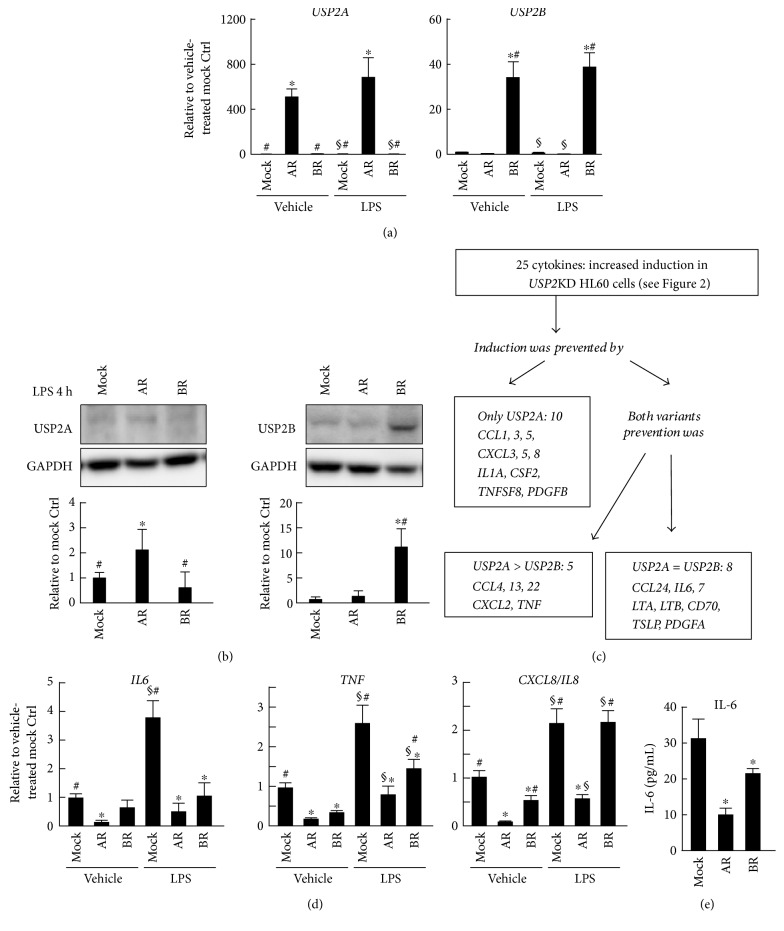
*USP2* variants modulate expression of proinflammatory cytokines. Cells were treated with PMA (30 nM; 1 d) and subsequently stimulated with LPS (2.5 *μ*g/mL) or vehicle alone for (a–d) 4 h or (e) 12 h. Transcript levels of (a) *USP2* splice variants and (d) cytokines were determined by qRT-PCR and are normalized according to *HPRT-1* transcript levels. (b) Western blot analysis of USP2 protein in the USP2 variant-restored cells stimulated with LPS. Signal intensities of the bands were measured and normalized to the level of GAPDH. Representative images are shown. (c) Schematic summary of cytokine expression analysis in the USP2 variant-restored cells. Four biological replicates were used for this analysis. (e) The IL-6 concentration in the supernatant of USP2 variant-restored cells was determined by ELISA. Data are presented as means ± SD of (a, d) six, (b) three, or (e) eight samples. Mock, *USP2*KD cells with a mock vector; AR, *USP2*KD cells with a *USP2A* expression construct; BR, *USP2*KD cells with a *USP2B* expression construct. ^∗^*P* < 0.05 versus mock-transfected control cells; ^#^*P* < 0.05 versus USP2AR cells; ^§^*P* < 0.05 versus vehicle-treated cells.

We next investigated possible modulatory roles of the USP2 splice variants, USP2A and USP2B, in LPS-elicited cytokine production. We infected *USP2*KD cells with a lentivirus expression vector encoding *USP2A* or *USP2B* (creating USP2AR and USP2BR cells, resp.) [29]. Even after LPS stimulation, USP2AR and USP2BR cells exclusively expressed USP2A and USP2B, respectively (Figures 3(a) and 3(b)). Overexpression of the USP2 splice variants significantly repressed the LPS-elicited induction of approximately 90% of cytokines (23 out of 25), which was potentiated by USP2 deficiency (Figure 3(c)). USP2AR and USP2BR cells exhibited a decrease in *IL6* transcripts (approximately 87% and 71%, resp.) relative to the mock-infected control cells following LPS stimulation (Figure 3(d)). Similarly, there was a decrease in the IL-6 concentration in the supernatant of USP2AR and USP2BR cells (Figure 3(e)). In addition, seven cytokines also showed comparable attenuation of cytokine induction by both splice variants (Figure 3(c)). In sharp contrast, the two USP2 splice variants demonstrated different roles in the expression of the other cytokines; USP2AR cells strongly repressed the expression of all 23 cytokines following LPS stimulation, whereas USP2BR cells exhibited either a lower (five cytokine transcripts including *TNF*) or negligible (10 cytokine transcripts including *CXCL8*) decrease. Therefore, USP2A in particular has the potential to repress the production of proinflammatory cytokines in macrophages after TLR4 activation.

### 3.4. Peritoneal Macrophages from *Usp2a* Tg Mice Alter the Expression of Proinflammatory Cytokines after LPS Stimulation

**Figure 4:**
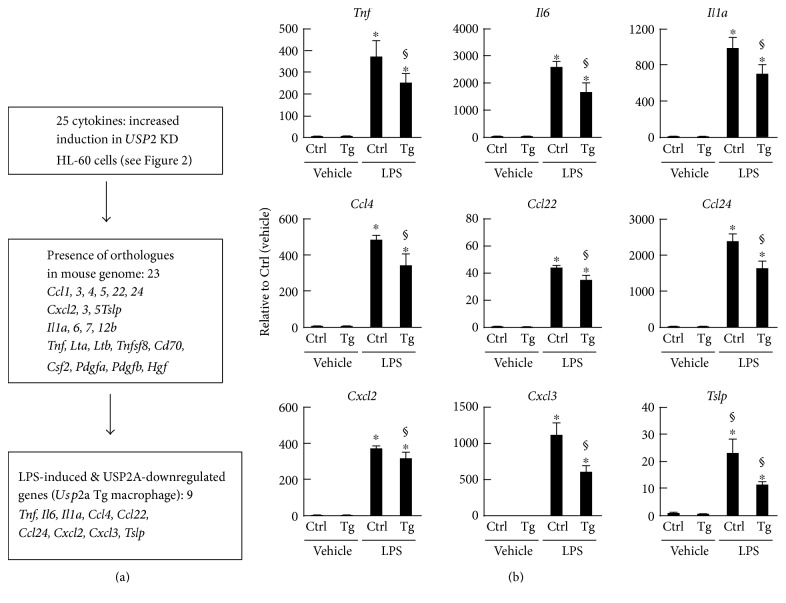
Cytokine expression in peritoneal macrophages isolated from *Usp2a* Tg mice. Peritoneal macrophages were isolated from *Usp2a* Tg mice and their littermates, then stimulated with LPS (5 *μ*g/mL) for 4 h. Transcript levels of *Usp2* splice variants and cytokines were determined by qRT-PCR analysis. (a) Schematic summary of the comprehensive cytokine expression analysis in *Usp2a* Tg macrophages. (b) Expression data of nine cytokine transcripts whose level was attenuated by the overexpression of *Usp2a*. Data were normalized according to the *HPRT-1* transcript level and expressed as means ± SD of five mice. ^∗^*P* < 0.05 versus vehicle-treated cells. ^§^*P* < 0.05 versus macrophages isolated from control mice.

To further evaluate the anti-inflammatory properties of USP2A in macrophages, we employed a different macrophage model. We measured cytokine gene expression in macrophages isolated from Tg mice ectopically expressing *Usp2a* under the control of the *FIRE* promoter [24, 29]. After treatment with LPS (5 *μ*g/mL) for 4 h, we measured the expression levels of cytokine transcripts in macrophages isolated from *Usp2a* Tg mice, as well as in littermate control mice. Of the 25 cytokines whose induction by LPS was potentiated in *USP2*KD cells (Figure 2(c)), 23 cytokines are encoded in the mouse genome (Figure 4(a)). Of these, 17 cytokines were induced in control macrophages by LPS treatment. Although *Usp2a* Tg macrophages also exhibited the induction of all cytokines, overexpression of *Usp2*a significantly repressed the induction of nine cytokines relative to control macrophages (Figures 4(a) and 4(b)). Therefore, USP2A modulates cytokine production not only in HL-60 cells, but also in peritoneal macrophages from Tg mice.

### 3.5. Ubiquitin Isopeptidase Activity of USP2A Is Crucial for the Inhibition of Cytokine Expression in HL-60 Cells

**Figure 5:**
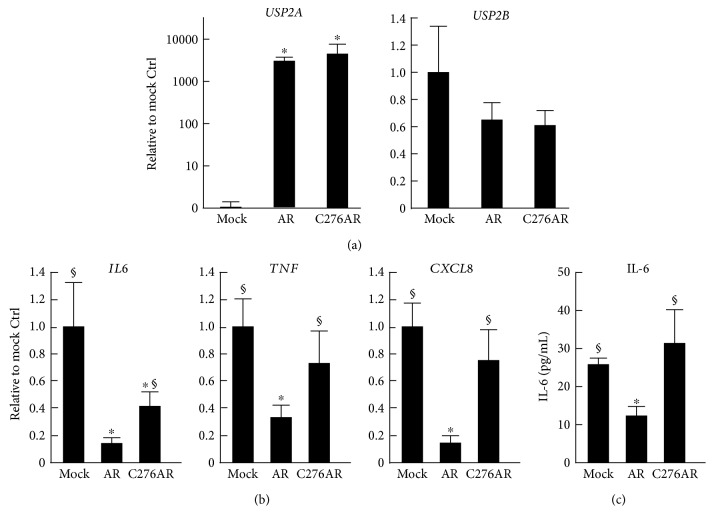
The isopeptidase region of USP2A is required for the downregulation of cytokine expression in macrophage-like cells. Wild-type and isopeptidase-deficient USP2A mutants were overexpressed in *USP2*KD cells. Cells were treated with PMA (30 nM; 1 d) and then stimulated with LPS (2.5 *μ*g/mL) for (a, b) 4 h or (c) 12 h. Transcripts for (a) *USP2* variants and (b) cytokines were assessed by qRT-PCR. Data were normalized according to *HPRT-1* transcript levels. (c) IL-6 concentration in the supernatant was determined by ELISA. Data are presented as means ± SD of (a, b) six or (c) eight samples. Mock, *USP2*KD cells with a mock vector; AR, *USP2*KD cells with a *USP2A* expression construct; C276AR, *USP2*KD cells with an isopeptidase-deficient *USP2A* mutant expression construct. ^∗^*P* < 0.05 versus mock-transfected control cells; ^§^*P* < 0.05 versus USP2AR cells.

We previously demonstrated that the C-terminal ubiquitin isopeptidase region of USP2A is crucial for the expressional control of chemokines under noninflammatory conditions [29]. We therefore investigated whether ubiquitin isopeptidase activity is also necessary for regulating proinflammatory cytokine expression after LPS stimulation. For this purpose, we utilized *USP2*KD cells ectopically expressing a peptidase-deficient USP2A mutant (C276AR cells; Figure 5(a)). As mentioned above, ectopic expression of USP2A strongly attenuated LPS-elicited cytokine induction (Figure 5(b)). In contrast, ubiquitin isopeptidase-deficient USP2 displayed limited or negligible effects when inducing *IL6*, *TNF*, and *CXCL8* after LPS stimulation. Similarly, the isopeptidase-deficient mutant failed to reduce the IL-6 concentration in the supernatant 24 h after LPS stimulation, whereas ectopically expressed USP2A clearly decreased IL-6 release from the macrophage-like cells (Figure 5(c)). Combined, these results show that the ubiquitin isopeptidase activity of USP2A contributes to repressing the LPS-elicited cytokine expression in macrophages.

### 3.6. TRAF6 and NF-*κ*B Are Not Targets of USP2 in LPS-Stimulated HL-60 Cells

**Figure 6:**
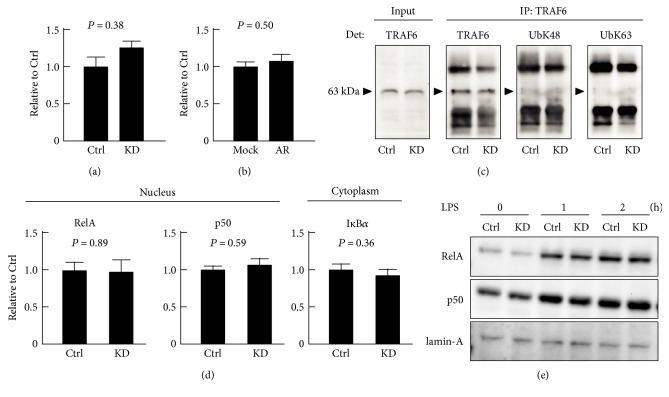
USP2A does not modulate the TRAF6-NF-*κ*B pathway in HL-60 cells. (a, c, d, e) PMA (30 nM; 1 d)-pretreated *USP2*KD (KD) and control (Ctrl) cells and (b) *USP2*KD cells infected with *USP2A* (AR) or empty construct (mock) were stimulated with LPS (2.5 *μ*g/mL) for (a–d) 1 h or (e) 0–2 h. Western blot analysis of TRAF6 protein levels in (a) *USP2*KD and (b) *USP2A*-restored cell. Values are normalized according to GAPDH levels. Data are presented as means ± SD of (a) nine or (b) seven samples. (c) K48- and K63-linked polyubiquitination of TRAF6 in *USP2*KD cells by immunoprecipitation-Western blot analysis. IP, antibody for immunoprecipitation; Det, antibody for detection. Arrowheads represent bands corresponding to the TRAF6 protein. Representative blot images of three independent experiments are shown. (d and e) Western blot analysis of nuclear RelA and p50 NF-*κ*B and cytoplasmic I*κ*B*α* in *USP2*KD cells. (d) NF-*κ*B and I*κ*B*α* protein levels are normalized to lamin-A and GAPDH levels, respectively. Data are presented as means ± SD of 16 (RelA and p50) or 12 (I*κ*B*α*) samples. (e) Representative Western blot images of NF-*κ*B components in the nucleus extracted from *USP2*KD and control cells after LPS stimulation.

To date, several studies have demonstrated that USP2 modulates the NF-*κ*B signaling pathway [30, 37, 38]. TRAF6, a signal transducer molecule associated with the TLRs and IL-1 receptor, is a potent target of USP2 [30, 39]. Therefore, we monitored TRAF6 protein levels after LPS stimulation. As shown in Figure 6(a), *USP2*KD cells did not show significant changes in the cellular TRAF6 content after LPS stimulation, although this experimental condition caused increased expression of cytokines in *USP2*KD cells (Figure 2). Moreover, USP2AR cells showed negligible changes in TRAF6 protein levels after LPS stimulation (Figure 6(b)).

K63-linked polyubiquitination has been proposed to modulate the interaction of signaling molecules, including TRAF6 [30, 40]. Next, we monitored the K63-linked, as well as the K48-linked polyubiquitination chain, in TRAF6 by immunoprecipitation-Western blot analysis (Figure 6(c)). The weak immune signal corresponding to K63-linked-ubiquitinated TRAF6 was detectable for both *USP2*KD and control cells after LPS stimulation. *USP2* knockdown did not influence K63-linked polyubiquitination in TRAF6. Similarly, K48-linked polyubiquitination was not affected by USP2 deficiency. Collectively, TRAF6 is unlikely to be a direct target of USP2 in HL-60 cells after LPS stimulation.

We then monitored the protein levels of nuclear NF-*κ*B components, RelA, and p50, and their cytoplasmic inhibitory protein, I*κ*B*α*, following LPS stimulation. As shown in Figures 6(d) and 6(e), compared to control cells, knockdown of *USP2* did not further affect the protein levels of NF-*κ*B nuclear components and cytoplasmic I*κ*B*α* 1-2 hours after LPS stimulation. Similar data were obtained 30 min after LPS stimulation (data not shown). In summary, the TRAF6-NF-*κ*B cascade does not determine the difference in cytokine production between *USP2*KD and control HL-60 cells.

### 3.7. USP2 Modulates Oct-1 and Oct-2 in LPS-Stimulated HL-60 Cells

**Figure 7:**
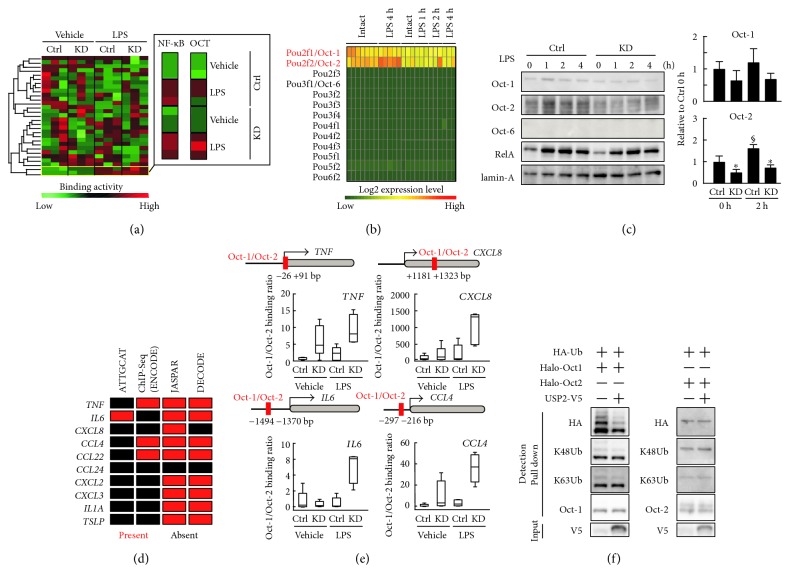
USP2 may regulate cytokine expression by polyubiquitination of Oct transcription factors in LPS-stimulated HL-60 cells. (a) DNA binding activity determined by bead array. Differentiated *USP2*KD and mock-transfected control (Ctrl) HL-60 cells were treated with LPS (2.5 *μ*g/mL) or vehicle alone for 1 h, and the nuclear fraction was subjected to analysis. A heatmap represents data from 29 transcription factors whose DNA binding activity increased during macrophage-like differentiation. NF-*κ*B and Oct data are also shown as enlarged pictures. (b) A heatmap showing an abundance of transcripts for POU family transcription factors in mouse macrophages. (c) Detection of Oct family proteins in *USP2*KD and control cells following LPS stimulation. Signal intensities of the bands were measured and normalized to lamin-A levels. Data are presented as means ± SD of five samples. Representative images are also shown. ^∗^*P* < 0.05 versus control cells ^§^*P* < 0.05 versus 0 h. (d) Schematic summary of Oct-1/2 binding sites in the 5′-proximal regions of the cytokine genes regulated by USP2. 5′-proximal Oct-1/2 binding sites were predicted by presence of the octamer motif, ATTGCAT, and Oct-2 ChIP signals (ENCODE database) and also surveyed using public prediction tools (JASPAR and DECODE databases). Ten cytokine genes tightly controlled by USP2 were analyzed. (e) The DNA binding ratio of Oct-1 to Oct-2 at the *TNF*, *CXCL8*, *IL6*, and *CCL4* promoters. ChIP-PCR was performed using five replicates. Data were normalized with those of input samples and represented as box-and-whisker plots. Oct-binding regions used for ChIP assays are depicted on each graph. (f) Detection of total K48- and K63-linked polyubiquitination on Oct proteins. Halo-tagged Oct-1 or Oct-2 and V5-tagged USP2A or mock plasmids with a HA-ubiquitin plasmid were introduced into HEK293FT cells for two days. Halo-tagged Oct proteins were pulled down and subjected to Western blot analysis. Input samples were also loaded as a reference. The HA-tag, K48- and K63-linked polyubiquitination chains (K48Ub and K63Ub), Oct-1, Oct-2, and the V5-tag were detected. Representative blot images are shown.

Since *USP2*KD HL-60 cells did not show changes in the nuclear accumulation of RelA and p50, other regulatory molecule(s) seem to be responsible for the augmented cytokine induction in *USP2*KD cells. To explore this, we measured the binding activities of 84 transcription factors using a bead array. In agreement with Figures 6(d) and 6(e), LPS increased the NF-*κ*B binding activity in *USP2*KD and control cells compared to the vehicle-treated cells (Figure 7(a)). Oct binding activity also increased after LPS stimulation and was relatively higher in *USP2*KD cells than in control cells. Since Oct proteins consist of eight POU transcription factors, we verified which Oct/POU transcription factor is dominantly expressed in macrophages using the public expression database RefDic. As previously documented [41], Oct-1 and Oct-2 are exclusively expressed in mouse macrophages regardless of LPS stimulation (Figure 7(b)). Western blot analysis clearly demonstrated that Oct-1 and Oct-2, but not Oct-6, were detectable in the nuclei of *USP2*KD and control cells (Figure 7(c)). After LPS stimulation, Oct-2 protein levels significantly increased (approximately 1.6-fold, *P* < 0.001 versus vehicle-treated cells) in control HL-60 cells, while nuclear Oct-1 protein level was very slightly affected (approximately 1.2-fold, *P* = 0.64 versus vehicle-treated cells). In *USP2*KD cells, Oct-2 protein levels were lower than mock-transfected control cells regardless of LPS stimulation. Similarly, Oct-1 tended to be downregulated in *USP2*KD cells. Therefore, USP2 potentiates nuclear accumulation of Oct-1 and Oct-2 proteins.

Because the expression of nine cytokines genes, namely *TNF*, *IL6*, *IL1A*, *CCL4*, *CCL22*, *CCL24*, *CXCL2*, *CXCL3*, and *TSLP*, were modulated by USP2 deficiency in HL-60 cells (Figure 2()), and overexpressed in HL-60 cells (Figure 3()) and isolated mouse macrophages (Figure 4()), we next surveyed the Oct-1 and Oct-2 binding sites in the proximal regions of the above cytokines and the *CXCL8* gene using public transcription factor binding site databases. ChIP sequence data from B cells deposited in ENCODE showed significant Oct-2 binding to the promoters of *TNF*, *CCL4*, and *CCL22* genes; however, the canonical octamer motif ATGCAAA was only present in the *IL6* promoter (Figure 7(d)). Moreover, either JASPAR or DECODE databases indicated that the 5′-proximal regions of nine out of 10 cytokine genes possess the Oct-1/Oct-2 binding sites. Thus, the Oct-1/Oct-2 binding sites are distributed to the proximal region of most of the cytokine genes, which are dominantly regulated by USP2.

Previous reports demonstrated that Oct-2 competitively inhibited Oct-1-elicited induction of the *iNOS/NOS2* gene [16]. Thus, we next assessed the promoter binding ratio of Oct-1 to Oct-2 in *USP2*KD and control cells. For this analysis, we selected four cytokines (*TNF*, *CXCL8*, *IL6*, and *CCL4*) based on the Oct-1/Oct-2 binding site analyses. The Oct-1/Oct-2 binding ratio at the *TNF*, *CXCL8*, and *CCL4* promoters was higher in *USP2*KD cells than in control cells and was significantly increased by LPS stimulation. Similarly, the Oct-1/Oct-2 binding ratio at *IL6* was remarkably increased in *USP2*KD cells after LPS stimulation; however, the increase was not observed in control cells. Taken together, USP2 deficiency induced an imbalance in Oct-1 and Oct-2 protein recruitment at the promoter regions of *TNF*, *IL6*, *CXCL8*, and *CCL4*.

It is possible that USP2 deficiency caused aberrant digestion of the polyubiquitin chain, resulting in remarkably reduced Oct-2 in the nucleus. To evaluate this, we examined whether USP2 potentially modulates K48-linked polyubiquitination in Oct proteins. Because endogenous K48- and K63-linked polyubiquitination of the Oct proteins is scarcely detected in HL-60 cells, we performed pull-down experiments using HEK293FT cells overexpressing USP2, ubiquitin, and Oct proteins. As shown in Figure 7(f), forced expression of USP2A failed to affect the total polyubiquitination of Oct-2, which was evaluated by a HA-tag-derived signal. Accordingly, ectopic expression of USP2A did not modify K48- and K63-linked polyubiquitination in Oct-2. In contrast, USP2-overexpressing cells showed a clear reduction of ubiquitinated Oct-1. Similarly, reduced K48- and K63-linked polyubiquitination chains were also evident on Oct-1 in USP2-overexpressing cells. Therefore, USP2 has the potential to modulate polyubiquitination of Oct-1, but not Oct-2.

## 4. Discussion

Macrophages are a major source of proinflammatory molecules such as cytokines, chemokines, and eicosanoids; hence, they are considered a critical component of inflammatory regulation [1]. In this study, we demonstrated that *USP2*KD promotes 25 of 104 cytokines in macrophage-like cells after LPS stimulation, while ectopic expression of *USP2* represses most cytokine production following this stimulation. These results indicate that the impact of USP2 on cytokines is extensive rather than restrictive in macrophage-like cells. Specifically, IL-6, IL-8, and TNF-*α* can contribute to the induction of hepatic acute phase proteins [8, 42], stimulation of fibroblast proliferation [8], promotion of neutrophil infiltration to septic lesions [9], and activation of the hypothalamic-pituitary adrenal axis [43]. USP2 thus has potential as a negative regulator of systemic inflammatory responses elicited by LPS-stimulated macrophages.

USP2 encodes two splice variants, USP2A and USP2B [26]. Although they share the same ubiquitin isopeptidase region on the C-terminus, their roles in cytokine expression differ; USP2A strongly repressed the expression of *TNF* and *CXCL8*, while the effect of USP2B was relatively weak or nonsignificant. Although our results do not rule out significant involvement of USP2B in cytokine repression, USP2A seems to be an effector variant repressing LPS-elicited cytokine production. The evidence accumulated thus far suggests that the two USP2 splice variants also have distinct roles in several other cell models, including skeletal muscle maturation [28], carcinogenesis of prostate cancer [44, 45], glucose metabolism in hepatocytes [27], and regulation of metabolic disease-associated genes in adipose tissue macrophages [29]. The varying roles of the USP2 splice variants seem to be attributable to structure differences in their N-terminal extensions. The part of the USP2A N-terminus that differs from USP2B consists of 258 amino acids, while that of the USP2B comprises only six amino acids [26]. Thus, USP2A is likely to be associated with certain scaffold proteins that USP2B is unable to bind. In support of this concept, our previous study revealed distinct cellular localization of USP2 splice variants in several types of cells; USP2A shuttles between the nucleus and cytoplasm, whereas USP2B resides only within the cytoplasm [29]. The different localization of these USP2 splice variants may contribute to their distinct roles in cellular responses.

In contrast to IL-8 and TNF-*α*, induction of eight cytokines was inhibited by both USP2A and USP2B to a similar extent. Half of these cytokines, namely CCL-24 (official gene symbol, *CCL24*), IL-7 (official gene symbol, *IL7*), lymphotoxin *β* (LT*β*; official gene symbol, *LTB*), and thymic stromal lymphopoietin (TSLP; official gene symbol, *TSLP*) were not induced in mock-transfected control cells after LPS stimulation. On the other hand, these cytokines account for 80% of the class in which USP2 deficiency is essential for LPS-elicited induction (Figure 2(c)). Considering the cellular localization of USP2 splice variants, these cytokines may be regulated by cytoplasmic protein(s), which are catalyzed by both USP2 variants. Interestingly, IL-7, TSLP, and LT-*β* are cytokines that mediate the development of lymphoid cells and organs [46–48]; therefore, the data suggests that USP2 is a key molecule in modifying lymphopoiesis through IL-7, TSLP, and LT-*β* expression.

In our previous study, *Usp2a* Tg mice exhibited minimal accumulation of USP2A proteins in their macrophages, although their *Usp2a* transcript levels had increased dramatically [29]. Small increases in USP2A protein levels may result in relatively weak expressional changes in metabolic disorder-associated genes in adipose tissue macrophages, followed by gradual changes in insulin sensitivity [24]. This implies that certain posttranscriptional or posttranslational mechanisms work to keep intracellular USP2 pools at a constant level in isolated macrophages. This marginal effect of the *Usp2a* transgene in isolated cells was also observed in the current study, whereby overexpression of USP2A in HL-60 cells repressed the induction of 25 cytokines, of which 23 were also encoded in the mouse genome. However, USP2A repressed only half of the cytokines in the macrophages isolated from *Usp2a* Tg mice. Therefore, from a clinical perspective, ways to efficiently sustain USP2A proteins in macrophages *in vivo* to prevent inflammatory disease are still needed.

Digestion of K63-linked polyubiquitination in TRAF6 by USP2A negatively regulates IL-1*β*- and Sendai virus-induced NF-*κ*B signaling in HEK293 and HCT116 cells [30]. Although TLR4-dependent signaling shares components, including TRAF6, with IL-1*β*- and virus-elicited signaling, we failed to detect any modification of K63-linked polyubiquitination in TRAF6 in our *USP2*KD HL-60 model. USP2A has been shown to exhibit positive effects on TRAF6-NF-*κ*B signaling in T-lymphocytes [39]; hence, the cell type may determine the consequences of USP2A-dependent signaling.

In contrast to negligible differences in NF-*κ*B binding activity between *USP2*KD and control cells, we found a slight increase in Oct binding activity in USP2-deficient conditions. Of the eight Oct proteins, only Oct-1 and Oct-2 are exclusively expressed in macrophages, implying certain roles of these Oct proteins in macrophage biology. In this report, we focused on the roles of Oct-1 and Oct-2 in USP2-modulated cytokine induction. A previous ChIP-seq analysis demonstrated that 50% of Oct-2 binding sites in a B cell line do not possess the ATTTGCAT octamer motif [14]. In agreement, we did not observe the octamer motif in the proximal regions of nine of 10 cytokines (cytokines in Figure 7(d) except for *IL6*), whereas most of the USP2-modulated cytokine genes have Oct-1/Oct-2 binding sites in the 5′-proximal region. Thus, unidentified transcriptional factors may associate with Oct-1 or Oct-2 and subsequently recruit to the promoters of the cytokine genes. Conversely, our in silico prediction failed to detect Oct-1/Oct-2 binding sites in the promoter region of *CCL24*, which is also controlled by USP2. Oct-1 and Oct-2 might be recruited to unidentified distal enhancer elements of the gene.

In this study, we found that USP2 showed a greater potentiation of nuclear accumulation of Oct-2 than Oct-1 after LPS stimulation. This observation suggests that USP2 affects the balance of Oct-1 and Oct-2 proteins. To support this idea, USP2 deficiency increased the DNA binding ratio of Oct-1 to Oct-2 in the *TNF*, *CXCL8*, *CCL4*, and *IL6* promoters, especially after LPS stimulation. Recently, Bentrari et al. [16] demonstrated that Oct-2 acts as a competitive inhibitor against Oct-1 at the *iNOS/NOS2* gene site by interfering with Pol II recruitment. Similar inhibition by Oct-2 may occur in the cytokine promoters in HL-60 cells. In our preliminary experiment, *USP2* knockdown did not affect *POU2F2* (official Oct-2 gene symbol) mRNA in HL-60 cells irrespective of LPS stimulation. This observation suggests that USP2 regulates the amount of endogenous Oct-2 at the protein level. Overexpression of USP2 did not cause modification of the K48-linked polyubiquitination of Oct-2 protein, suggesting that USP2 is unlikely to modulate Oct-2 digestion in a direct manner. Certain indirect mechanisms may modulate Oct-2 protein levels. OBF-1, a coactivator of Oct-1 and Oct-2, is controlled at the protein level by SIAH1 ubiquitin ligase [49]. Similar mechanisms may contribute to Oct-2 degradation; USP2 might modify K48-linked polyubiquitination of certain ubiquitin ligases, promoting an increase of Oct-2 in HL-60 cells after LPS stimulation.

In contrast to Oct-2, Oct-1 protein levels were only slightly decreased (approximately 0.58-fold) by USP2 deficiency, although the K48-linked polyubiquitin chain was significantly digested in *USP2A*-overexpressing cells (Figure 7(f)). Overexpression of USP2 also promoted the digestion of the K63-linked polyubiquitin chain of Oct-1; hence, K63-linked polyubiquitination might influence the stability of Oct-1. A previous study demonstrated that the K63-linked polyubiquitin chain of Imd, a *Drosophila* homologue of RIPK, protects the protein from proteasome-dependent degradation [50]. Similar mechanisms might contribute to the limited digestion of Oct-1 in *USP2*KD cells. In addition, USP2 may also impede Oct-1-dependent transcription by disturbing protein-protein interactions. In support of this idea, previous reports have demonstrated that several transcription factors exert transcriptional activation by interacting with the Oct-1-Pol II relationship [51].

## 5. Conclusion

Given that the longer splice variant of USP2 negatively controlled the expression of approximately 25% of 104 cytokines, it accounts for 80% (20 of 25) of LPS-elevated cytokines, where USP2 is a global repressor of cytokines in HL-60 macrophages following TLR4 activation. In addition, the isopeptidase domain of USP2 is required for the downregulation of global cytokine expression. Interestingly, we identified Oct-1 and Oct-2 as novel targets of USP2 in the inflammatory responses contributed by macrophages. Moreover, we found that USP2 regulates proportional changes when recruiting Oct-1 and Oct-2 on cytokine promoters. Future studies will verify how the modulation of Oct proteins by USP2 is involved in cytokine induction.

## Figures and Tables

**Table 1:** Sequences of qRT-PCR primers and probes. Sequences of primers and probes used for qRT-PCR analysis are shown. Hs and Ms represent *Homo sapiens* and *Mus musculus*, respectively.

Species	Gene	Sequences
Hs	*IL6*	Fwd: 5′-CTCAGCCCTGAGAAAGGAGA-3′
		Rev: 5′-TTTCAGCCATCTTTGGAAGG-3′
Hs	*CXCL8*	Fwd: 5′-CCCCAAATTTATCAAAGAACTGAGAG-3′
		Rev: 5′-AAACTTCTCCACAACCCTCTGC-3′
		Probe: 5′-TGGACCACACTGCGCCAACACAGAA-3′
Hs	*TNF*	Fwd: 5′-TCAGCCTCTTCTCCTTCCTG-3′
		Rev: 5′-GCCAGAGGGCTGATTAGAGA-3′
Hs	*HPRT1*	Fwd: 5′-GGTCAGGCAGTATAATCCAAAGATG-3′
		Rev: 5′-AACAAAGTCTGGCTTATATCCAACAC-3′
		Probe: 5′-TCGTGGGGTCCTTTTCACCAGCAAGC-3′
Ms	*Usp2A*	Fwd: 5′-TATGGCACCTACACCCCTTC-3′
		Rev: 5′-CCCCTGTCACAGTCCAGAAT-3′
Ms	*Usp2B*	Fwd: 5′-GCGTACCTCCTACACGGTGA-3′
		Rev: 5′-TCTTGGCTTTGTTGAGCAGA-3′
Ms	*Il6*	Fwd: 5′-GTTCTCTGGGAAATCGTGGA-3′
		Rev: 5′-TTCTGCAAGTGCATCATCGT-3′
Ms	*Tnf*	Fwd: 5′-CCACCACGCTCTTCTGTCTA-3′
		Rev: 5′-AGGGTCTGGGCCATAGAACT-3′
Ms	*Il1a*	Fwd: 5′-CGCTCAAGGAGAAGACCAG-3′
		Rev: 5′-AAATGAGGTCGGTCTCACTACC-3′
Ms	*Ccl4*	Fwd: 5′-CCCACTTCCTGCTGTTTCTC-3′
		Rev: 5′-GCTGCTCAGTTCAACTCCAA-3′
Ms	*Ccl22*	Fwd: 5′-TATGGTGCCAATGTGGAAGA-3′
		Rev: 5′-AGGTCCTCCTCCCTAGGACA-3′
Ms	*Ccl24*	Fwd: 5′-CTGTGACCATCCCCTCATCT-3′
		Rev: 5′-TATGTGCCTCTGAACCCACA-3′
Ms	*Cxcl2*	Fwd: 5′-AGTTTGCCTTGACCCTGAAG-3′
		Rev: 5′-CTTTGGTTCTTCCGTTGAGG-3′
Ms	*Cxcl3*	Fwd: 5′-TGGTCAAGAAGTTTGCCTCA-3′
		Rev: 5′-GGATGGATCGCTTTTCTCTG-3′
Ms	*Tslp*	Fwd: 5′-AGAGAAGCCCTCAATGACCA-3′
		Rev: 5′-TTCTGGAGATTGCATGAAGG-3′
Ms	*Hprt1*	Fwd: 5′-TCATTATGCCGAGGATTTGG-3′
		Rev: 5′-ACTTTTATGTCCCCCGTTGA-3′

**Table 2:** Sequences of CHART-PCR primers. Sequences of primers used for CHART-PCR analysis with the SYBR green method are shown.

Gene	Sequences
*CXCL8*	Fwd: 5′-GACTCAGGTTTGCCCTGAGGGGATG-3′
	Rev: 5′-GCTTGTGTGCTCTGCTGTCTCTGAA-3′
*IL6*	Fwd: 5′-CCAGCCATCCTCCCCCATTTTCATT-3′
	Rev: 5′-CAGGCTGAAACCAGACCCTTGCACA-3′
*TNF*	Fwd: 5′-TGAATGATTCTTTCCCCGCCCTCCT-3′
	Rev: 5′-CACGTCCCGGATCATGCTTTCAGTG-3′
*HBB*	Fwd: 5′-AAGCCAGTGCCAGAAGAGCCAAGGA-3′
	Rev: 5′-CCCACAGGGCAGTAACGGCAGACTT-3′
*GAPDH*	Fwd: 5′-ACCTCCCATCGGGCCAATCTCAGTC-3′
	Rev: 5′-GGCTGACTGTCGAACAGGAGGAGCA-3′
*RPS6*	Fwd: 5′-TGCAAAGTGCCTGGGACAGAAGTGG-3′
	Rev: 5′-CGCAGGTCACATAGGCGCTTTCAGT-3′

**Table 3:** Sequences of ChIP-PCR primers. Sequences of primers used for ChIP-PCR analysis with the SYBR green method are shown.

Gene	Sequences
*TNF*	Fwd: 5′-AAAGGCAGTTGTTGGCACAC-3′
	Rev: 5′-TGGCGTCTGAGGGTTGTTTT-3′
*CXCL8*	Fwd: 5′-AAGTTCCAGGTGTTAGGATTACAGT-3′
	Rev: 5′-ATTCCTTAAGTCAGGCATAAAGTCT-3′
*IL6*	Fwd: 5′-AGAGGACCACCGTCTCTGTT-3′
	Rev: 5′-CAGTGACCTCTGTTGGGCAT-3′
*CCL4*	Fwd: 5′-GCCACTTGTAGCAGGTGTGA-3′
	Rev: 5′-CAAAGGCAGTGACCGAGACT-3′
